# Bowman’s layer and corneal thickness in health and disease

**DOI:** 10.1136/bmjophth-2025-002167

**Published:** 2025-05-22

**Authors:** Yaochun Shen, Yalin Zheng, Alfredo Borgia, Matteo Posarelli, Rose Herbert, Tom Sharp, Luca Pagano, Vito Romano, Andrea Madden, Alexander Undan, Stephen B Kaye

**Affiliations:** 1 Electrical Engineering and Electronics, University of Liverpool, Liverpool, UK; 2 Department of Eye and Vision Science, University of Liverpool, Liverpool, UK; 3 Corneal and External Disease, Royal Liverpool University Hospital, Liverpool, UK; 4 Royal Liverpool University Hospital, Liverpool, UK; 5 Cornea, Royal Liverpool University Hospital, Liverpool, UK; 6 Ophthalmology, Royal Liverpool and Broadgreen Hospitals NHS Trust, Liverpool, UK; 7 Ophthalmology, Humanitas Research Hospital, Milan, Italy; 8 University of Brescia, Brescia, Italy

**Keywords:** Anatomy, Cornea, Imaging

## Abstract

**Purpose:**

To investigate central Bowman’s layer thickness (BT) in relation to central corneal thickness (CCT) and curvature, and epithelial thickness in healthy and disease corneas.

**Methods:**

Patients with keratoconus (KC), corneal dystrophies (CD) and healthy controls (HC) were included. Linnik and Mirau versions of an ultra-high axial resolution line field spectral domain optical coherence tomography device were used to image the cornea, in addition to commercially available devices. A supervised automated segmentation process was used to extract the quasi-point thickness of Bowman’s layer.

**Results:**

62 participants: 24 with KC, 20 with CD and 18 HC were included. Mean central BT was 15.41 µm (SD 0.49; min-max: 12.28–19.54) in HC, 14.27 µm (SD:0.43; min-max: 11.22–18.25) in KC and 15.65 µm (SD 0.64; min-max: 12.42–20.06) in CD (mainly Fuchs CD). Patients with KC had thinner central BT than those with CD (p=0.03), but not compared with HC (p=0.13). Central BT was significantly associated with CCT (p<0.01), being on average 3% of CCT. The ratio of BT to CCT was independent of diagnosis (CD 0.028, HC 0.030, KC 0.028, p=0.88), age (p=0.23), sex (p=0.67), Kmax (p=0.77) or epithelial thickness (p=0.72).

**Conclusions:**

Over sample populations of healthy, keratoconic and dystrophic corneas, central BT was consistently associated with corneal thickness and was independent of age, sex, Kmax, and epithelial thickness.

**Trial registration number:**

ISRCTN40558.

**WHAT IS ALREADY KNOWN ON THIS TOPIC**

- Bowmans’ layer has been measured in vivo using non-commercial systems. It has not, however, been measured in relation to corneal thickness.

**WHAT THIS STUDY ADDS**

- There is a relatively constant relationship between central Bowman’s layer and corneal thickness in healthy corneas, keratoconus and several types of corneal dystrophies including Fuchs endothelial corneal dystrophy.

**HOW THIS STUDY MIGHT AFFECT RESEARCH, PRACTICE OR POLICY**

- The relatively constant relationship between central Bowman’s layer and corneal thickness offers a clinical measure for assessing corneal status.

## Introduction

 Bowman’s layer is an acellular layer lying between the corneal epithelium and the stroma composed of randomly woven fibrils. Although the role of Bowman’s layer remains unclear, it is affected by diseases of the cornea.^1^,^7^ It has been shown that Bowman’s layer is actively maintained by ongoing epithelial-stromal interactions and may be compromised by diseases affecting these layers. For example, disruption of Bowman’s layer has been seen in keratoconus (KC).^1^,^4^ Using electron microscopy, thinning of Bowman’s layer has been suggested to occur before thinning of the stroma,^5^ but the evidence, however, is unclear. In the advanced stages of Fuchs endothelial corneal dystrophy (FED), corneal fibroblasts enter Bowman’s layer.^6 7^ The changes that have been described in Bowman’s layer have largely been reliant on histological studies and, as a consequence, are skewed towards cases with more advanced disease.

Optical coherence tomography (OCT) is able to measure Bowman’s layer thickness (BT) in vivo to an accuracy down to 0.3 µm SDs (ie, approximately a 1.2 µm 95% CI for a single measurement),^8 9^ although this value is likely inflated by genuine local spatial variance in thickness. The anterior boundary of Bowman’s layer with the cellular epithelium and the suboptical resolution epithelial basement membrane shows a much clearer transition than the posterior surface and corneal stroma.

There is limited information on measuring Bowman’s layer in vivo and whether BT varies according to other corneal parameters. Shousha *et al.,* measured BT in the vertical direction over a 9 mm range, from the superior to inferior part of the cornea, and noted that in KC, Bowman’s layer was thinner in the inferior cornea.^10^ This was supported by Pircher *et al.*, who mapped the BT in both lateral dimensions.^11^ These studies, however, did not determine if the changes in BT differ and/or are concurrent with changes in the corneal stroma, particularly as in KC, thinning of the inferior cornea precedes the superior cornea.^12^

This type of information may help determine the role of Bowman’s layer and in which conditions and at what stage it becomes affected. To investigate, therefore, if changes in Bowman’s layer are distinct from those of the stroma, we developed a LiveOCT^8 13^ device, with ultrahigh axial imaging resolution (axial image PSF FWHM of 1.7 µm in corneal tissue (n_G_=1.387)^14 15^ to measure Bowman’s layer. This OCT system provides semiautomatic imaging segmentation over a 450 µm length to output a mean quasi-point BT with a repeatability of 0.3 µm SD.^8^ We used this system to investigate in vivo the relationships between central BT, central corneal thickness (CCT), corneal curvature (Kmax) and epithelial thickness in healthy participants (HC) and in patients with KC and corneal dystrophies (CD).

## Methods

A prospective study was undertaken between 2021 and 2023 in which patients with KC and CD were invited to participate online supplemental table 1. Healthy participants with no history of ocular diseases were also included as controls. Patients who had previous surgery, for example, refractive surgery, corneal crosslinking, nystagmus, inability to fixate on the target or the presence of other eye diseases were excluded. The primary endpoint was the measurement of the central BT in patients with KC and CD, and in HC.

### Imaging methods

The study included two variants of the LiveOCT device: the Mirau variant (described in detail in a previously published article^8^) and the more conventional Linnik variant (no Mirau interferometer) which has an added Linnik reference arm at the cube beam splitter.^8 13^ The light emissions at the location of the subject’s eye (near field) were within IEC 60825-1:2014 class 1 limits.^8^

The Linnik OCT device has approximately twice the working distance of the Mirau OCT device, while the latter promises a more compact design, integrating the imaging lens and interference optics into a single component. Although we have shown that each variant produces identical information (online supplemental table 1), we wanted to determine if both could be used in clinical practice. As shown in the online supplemental table 1, both devices have nearly identical performance specifications (eg, imaging depth, axial and lateral resolution, frame rate) since they use the identical light source, spectrograph and cameras. Participants were, therefore, imaged with both devices at each visit, with a minimum of three captures per eye per device, and the best image of Bowman’s layer, whether from the Linnik or Mirau, was used to measure BT.

Image data consisted of up to 50 B-Scan frames (frames below a signal threshold were automatically screened out), automatically (axial direction only) correlated and averaged.^8^ Segmentation was semiautomatic, being supervised by one of three trained clinicians with some overlap to allow comparison between supervisors. In the first step, the supervisor would confirm that the averaged image was suitable for segmentation (ie, it showed Bowman’s layer). The reflective boundaries of the tear film surface, epithelium-Bowman’s interface (epithelial basement membrane) and Bowman’s layer to stromal boundaries were iteratively segmented using a graph search method with supervisors blocking incorrect paths and selecting the paths that corresponded to the desired interfaces.^8 13 15^ The algorithm identified multiple low energy paths, and some other low paths across the images typically existed which did not relate to or affect the desired interface results. After the segmentations had been done, the quality of the image and segmentation results were graded (1–definitely not correctly interpretable, 2–probably not correctly interpretable, 3–moderate, 4–good, 5–exceptional). Only results scoring 3–5 were used in the subsequent analyses. Failure was defined as not being able to identify the boundaries of Bowman’s layer. The best image of central Bowman’s layer, therefore, whether from the Linnik or Mirau, was used to measure BT.

In order to capture and measure CCT (not apical in patients with KC) and curvature as Kmax, patients underwent imaging with Schiempflug (Oculus Pentacam, Oculus, Wetzlar, Germany). Images were also taken with a Heidelberg Spectralis (Heidelberg Engineering, Germany), Tomey CASIA2 (Tomey Corporation, Nagoya, Japan) and Heidelberg Anterion (Heidelberg Engineering, Heidelberg, Germany) to determine if Bowman’s layer could be measured with these devices.

### Analysis

The data for one eye of each subject at one visit were used for analysis. Statistical analysis was undertaken using SPSS (V.27). Analysis of variance with post hoc Tukey was used to compare variables across groups. Variables that were continuous and normally distributed were entered into a general linear model with sex and diagnosis as fixed and random factors. A p<0.05 was considered to be statistically significant after adjustment for multiple tests.

## Results

62 participants: 24 with KC (mean±SD age 32±9 years; female to male ratio 4:20), 20 with CD (age 69±14 years; female to male ratio 12:13, comprising 17 patients with FED, 1 with granular-lattice, 1 with posterior polymorphous dystrophy and 1 with iridocorneal endothelial dystrophy) and 18 HC (age 40 years±13, female to male ratio 9:9) were included who had image segment scores greater than 3. 28 subjects were excluded (9 CD, 3 HC, 16 KC) as they either were found to have had previous surgical interventions, for example, cross-linking, or had image segment scores not greater than 3 using the LiveOCT devices on at least one of the three visits.

### Measurement of Bowman’s layer

**Figure 1:**
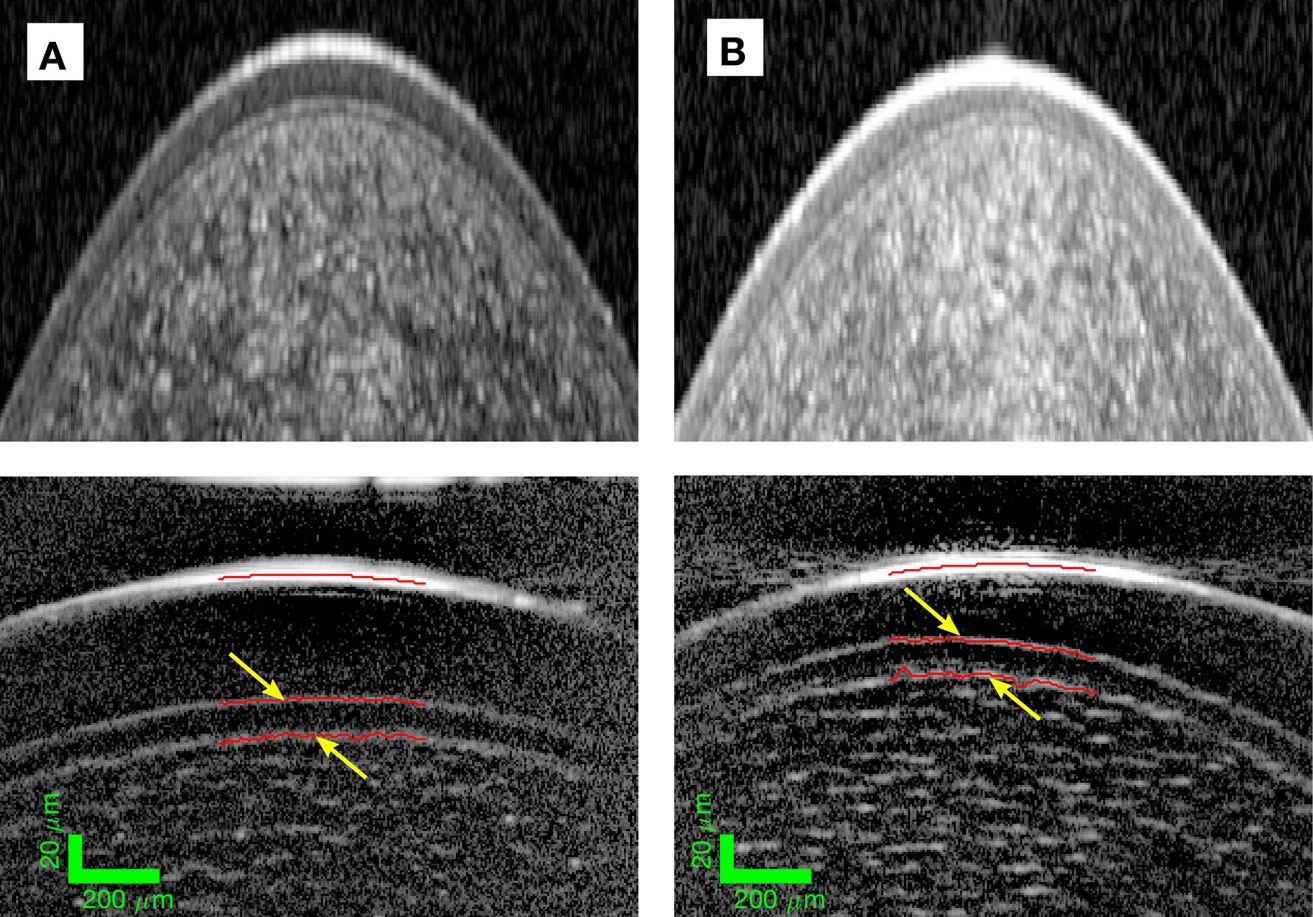
Imaging and measurement of Bowman’s layer. (**A**) (Left) Bowman’s layer is visible in the Heidelberg Spectralis image (top) but is better resolved by LiveOCT (bottom) in a healthy cornea with thickness semiautomatically segmented (red lines and yellow arrows). (**B**) (Right) Bowman’s layer is just visible in the Heidelberg Spectralis image (top) but is well resolved by LiveOCT (bottom) in a patient with KC with thickness semiautomatically segmented (red lines and yellow arrows). KC, keratoconus.

The interface between the epithelium and Bowman’s layer was found for all subjects to be relatively hyper-reflective (figure 1) and smooth as previously described with the LiveOCT.^8^ We were not able to reliably discern and measure Bowman’s layer with the Heidelberg Spectralis, Tomey CASIA2 and Heidelberg Anterion devices and, therefore, measured Bowman’s layer only with the LiveOCT devices (figure 1).

Bowman’s layer epithelial sits on the epithelial basement membrane, which is much thinner than enabled by the system’s axial resolution and is likely reflective in its own right. In contrast (figure 1), the brightness of the Bowman’s layer—stromal interface is less bright and not as easy to define. We have shown that the apparent interface segmented is much rougher in all cases.^8^

### Quality of image segmentation

Online supplemental figure 1 shows the highest quality score of segmentation given per eye per visit, indicating the reliability of LiveOCT to obtain central BT. Overall, for each eye, 58.8% of visits provided reliable central BT while 15.3% did not produce any meaningful result. Therefore, images with individual segmentation that scored less than 3 were excluded and average values were weighted to give prominence to the better-quality images (weighting factors score 3–weight 1, S4–W2, S5–W3). Reliable layer thickness measurement requires high-quality B-scan images in which the boundaries of Bowman’s layer are well-defined and can be accurately measured using the image segmentation algorithm. In KC patients, approximately 14.3% of images had a segmentation score of 2 or lower, as scarring in the cornea made it difficult to clearly image and define the layer boundaries. For other corneal conditions, we observed that around 18.6% of images had a score of 2 or lower, although the underlying cause requires further investigation.

### Central BT

Central BT was 15.41 um (SD 0.49; min-max: 12.28–19.54) in HC, 14.27 µm (SD:0.43; min-max:11.22–18.25) in KC, and 15.65 µm (0.64; min-max:12.42–20.06) in patients with CD (table 1).

**Table 1:** Corneal measurements, age and sex in HC, KC and CD

	Age (year)	BT (μm)	ET (μm)	CCT (μm)	Kmax (D)	BT/ET	BT/CCT
HC	40 (13)	15.41 (0.49)	52.72 (1.97)	539.72 (33.32)	43.73 (1.70)	0.31 (0.07)	0.03 (0.006)
KC	32 (9)	14.27 (0.43)	48.48 (7.81)	507.58 (37.54)	51.66 (8.43)	0.30 (0.06)	0.028 (0.003)
CD	69 (14)	15.65 (0.64)	52.39 (6.91)	564.44 (56.10)	45.88 (2.34)	0.30 (0.05)	0.028 (0.004)

Mean and SD of central BT, ET, CCT, Kmax, ratio of BT/ET and ratio of central BT to CCT (BT/CCT).

BT, Bowman’s layer thickness; CCT, central corneal thickness; CD, corneal dystrophies; ET, epithelial thickness; HC, healthy control; KC, keratoconus; Kmax, maximum keratometry.

**Figure 2:**
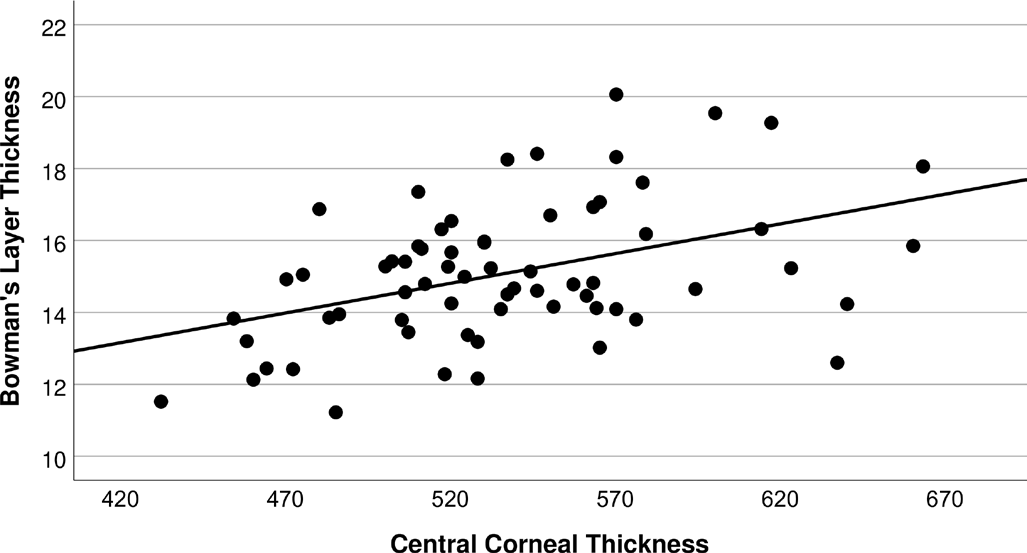
Linear association between central Bowman’s layer thickness and central corneal thickness (p<0.01, R^2^=0.18).

**Figure 3:**
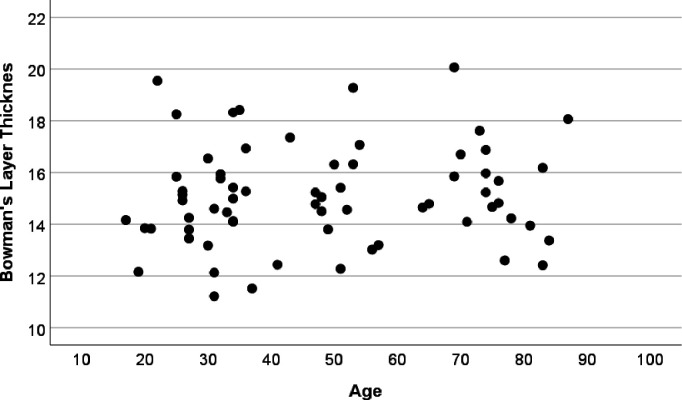
Bowman’s layer thickness and age. No significant association between Bowman’s layer thickness and age within the range included for the respective conditions (p=0.45).

There was a significant difference in BT between patients with KC and CD (p=0.03) but not between HC and KC (p=0.13) or HC and CD (p=0.91). There was a significant linear association (18%) between central BT and CCT (p<0.01 figure 2), but not with age (p=0.07, figure 3), diagnosis (p=0.35), sex (p=0.22), Kmax (p=0.52) or epithelial thickness (p=0.74). The ratio of BT to CCT was independent of age (p=0.23), sex (p=0.67), Kmax (p=0.77), epithelial thickness (p=0.72) or diagnosis (CD 0.028, HC 0.03, KC 0.028, p=0.88). Central epithelial thickness was significantly associated with Kmax, (p<0.001) but not CCT (p=0.70), BT (p=0.74), sex (p=0.71), age (p=0.86) irrespective of diagnosis (p=0.97).

### Ratio of BT to CCT

The ratio of central BT to CCT was independent of diagnosis (p=0.85), age (p=0.72), sex (p=0.21), Kmax (p=0.53) and epithelial thickness (p=0.93) (figures3  4).

**Figure 4:**
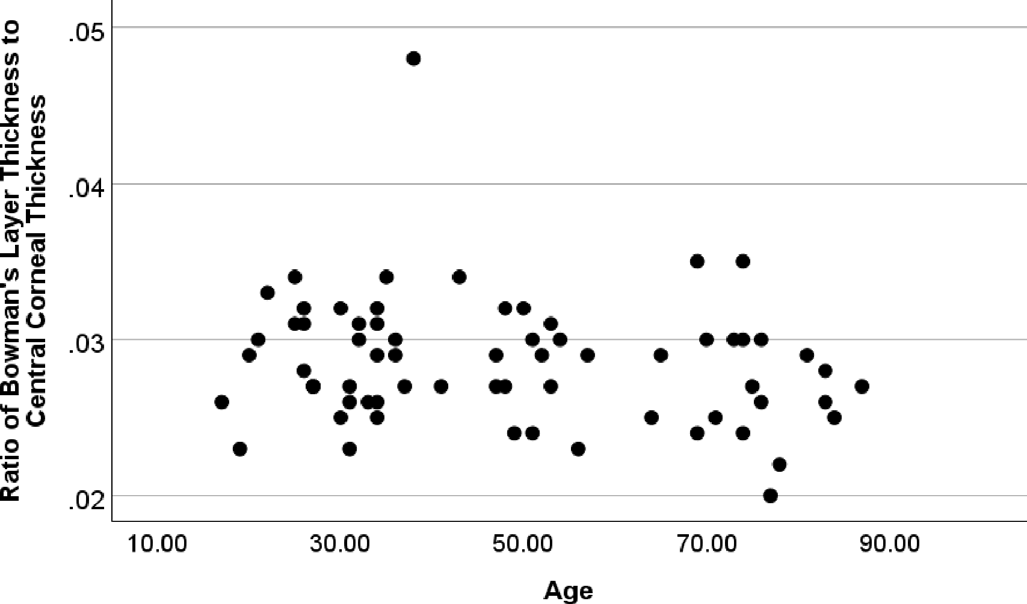
Ratio of central Bowman’s layer thickness to central corneal thickness and age. No significant association between the ratio of Bowman’s layer to central corneal thickness and age within the range included for the respective conditions (p=0.72).

## Discussion

In this study, we provide a method of systematically measuring central BT using image segmentation. This is effectively a de facto single point measurement with the 450 µm segmentation profile lengths (shorter than the scales thickness variations linked to KC shown by previous groups). To evaluate the impact on lateral location of the repeatability of the measurements, with every capture the LiveOCT system collected a photograph of the pupil from a camera fixed within the optical enclosure. By simple pupil tracking analysis, within that photograph, the relative (uncalibrated) lateral position of each capture for an eye by each device on each visit can be measured. This high level of quasi-random local variation of BT can be seen in the results of Shousha *et al.,*^10^ and our previous study.^8^

In our study, we demonstrated that central BT was significantly associated with CCT among all the different variables we considered (diagnosis, sex, age, Kmax and epithelial thickness). Indeed, we found that the ratio of BT to CCT was relatively constant and was independent of diagnosis (HC, KC or CD), or other corneal parameters (within the range of disease included for in these conditions). This relationship between central BT and CCT suggests that conditions such as KC, which affect the corneal stromal thickness, correspondingly affect the BT. This may reflect similarities in the composition of these layers and the corresponding effects of disease on their common components rather than any codependency. For example, it is evident in KC that disease progression leads to the thinning of the cornea (stroma) and an increase in corneal curvature concurrent with thinning of the overlying epithelium.^1 16^ Xu *et al.*, using ultra-high axial resolution OCT, observed a pattern of thinning in the central and inferior regions of Bowman’s layer in KC eyes.^17^

Although it has been shown by in vivo confocal microscopy that BT decreases with age,^18^ it was not clear if this was separate from concurrent changes in the corneal stroma. Although we could not find any significant association between BT and age in either healthy, KC or CD, this may reflect our sample size and age range in the healthy group. We intended to analyse potential longitudinal changes in BT over three visits, but there were too few subjects that had reliable BT measurements at all of the visits and that could be verified as having the same corneal location of the measurement. We, therefore, only included measurements of one eye at one of the visits. Further engineering improvements could, therefore, be made to LiveOCT, particularly in relation to optics enclosure stability and increased automation of image capture/alignment. There are limitations in our study in defining the boundaries of Bowman’s layer. This was undertaken using a semiautomatic segmentation process. This introduces some subjectivity, but to minimise bias, we used three observers to select paths that corresponded to the interfaces. Although we attempted to always align the location of the centre of Bowman’s layer and the cornea, because measurement of BT and CCT was undertaken using different devices, we could not be certain of alignment.

Although we provide a data set to infer a relationship between BT and CCT and other measures, a larger area or vertical profile length substantially longer than 450 mm would provide more clinical value. Longitudinal studies in health and disease measuring Bowman’s layer and other cornea parameters may help to further understand these relationships. Our current data suggest that an alteration in the ratio or homeostasis between BT and corneal thickness may indicate a disease or event that primarily affects one of these layers disproportionately. As clinical imaging of Bowman’s layer becomes more readily available, the given range of values for BT and reference of BT to CCT may serve as an index for detecting disease affecting either layer disproportionately.

## Supplementary material

10.1136/bmjophth-2025-002167 online supplemental table 1

10.1136/bmjophth-2025-002167 online supplemental figure 1

10.1136/bmjophth-2025-002167 online supplemental file 1

## Data Availability

Data are available on reasonable request.
