# Supplementary material for: Bowman’s layer and corneal thickness in health and disease
Source: BMJ Open Ophthalmol. 2025 May 22;10(1):e002167. doi: 10.1136/bmjophth-2025-002167 (PMC12104951; doi:10.1136/bmjophth-2025-002167)
Supplement: online supplemental table 1 [file bmjophth-10-1-s001.docx]

**Supplement Table 1. Comparison of the Linnik and Mirau devices used in this study**

| Device | **Linnik Device** | **Mirau Device** |
| --- | --- | --- |
| Light source wavelength (nm) | 700nm—1000nm | 700nm—1000nm |
| Image (A-Scan) depth (nG.mm) | 1.23 | 1.23 |
| B-Scan length (mm) | 2.29 | 2.29 |
| Axial resolution (nG.µm) | 2.4 | 2.4 |
| Lateral resolution (µm) | ~20 | ~20 |
| Axial image rate (k A-Scans/s) | 204.8 | 204.8 |
| Single frame sensitivity (dB) | 79 | 77 |
| Single frame dynamic range (dB)  (Glass interface signal / empty standard deviation) | 65 | 63 |
| Integration time (µs) | 250 | 500 |
