# Supplementary figures and images for: Bowman’s layer and corneal thickness in health and disease
Source: BMJ Open Ophthalmol. 2025 May 22;10(1):e002167. doi: 10.1136/bmjophth-2025-002167 (PMC12104951; doi:10.1136/bmjophth-2025-002167)

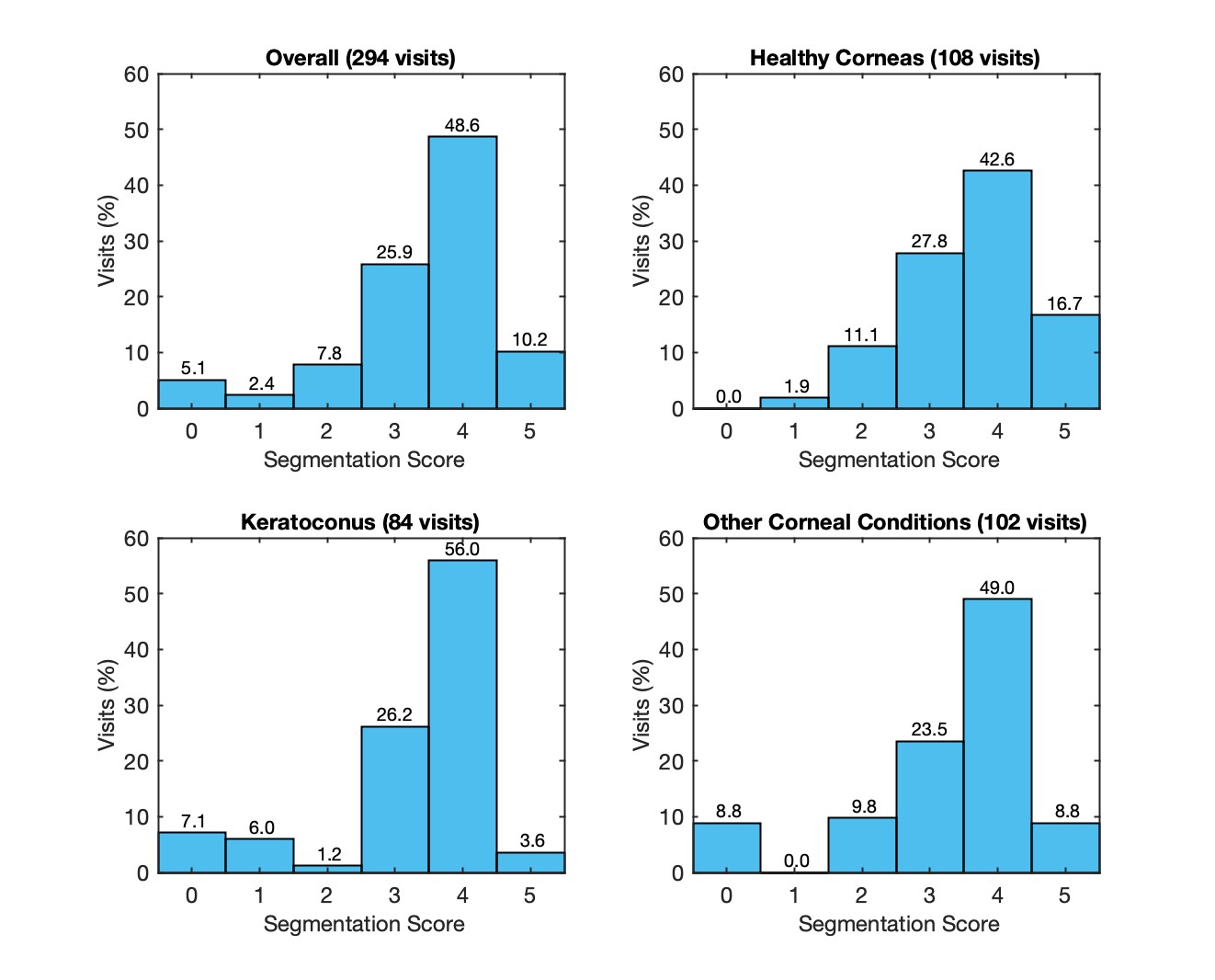

Supplement: online supplemental figure 1 [file bmjophth-10-1-s002.jpg]
