# Supplementary material for: Bowman’s layer and corneal thickness in health and disease
Source: BMJ Open Ophthalmol. 2025 May 22;10(1):e002167. doi: 10.1136/bmjophth-2025-002167 (PMC12104951; doi:10.1136/bmjophth-2025-002167)
Supplement: online supplemental file 1 [file bmjophth-10-1-s003.docx]

| Date | Version | Changes |
| --- | --- | --- |
| 03.12.2020 | 0.1 | Original draft sent to sponsorship committee |
| 04.02.2021 | 0.2 | Changes to study monitoring, patient experience, patient pathway, consenting children, duration of D1 and D2,and sample size calculation |
| 25.03.2021 | 0.3 | Changes on AE reporting examples of AEs, outcomes, and monitoring plan |
| 12.04.2021 | 0.4 | Changes to section 14. Spreadsheets locked every 3 months, missing data |
| 27.04.21 | 1.0 | Accepted by sponsorship committee |
| 27.07.21 | 1.1 | Response to NRES comments – clarification of age |
| 09.08.21 | 2.0 | Version 1.1 approved by Ethics committee so changes accepted and version number updated |
| 20.09.21 | 2.1 | Changes in response to MHRA queries (ref to ‘not over 100 years old’ removed and section 5 and 8 updated to reflect 3 tests with each LiveOCT device at EACH visit, ) |
| 30.09.21 | 2.2 | Changes in response to MHRA queries – 1) clarification of how the primary endpoint relates to the sample size calculation (section 13) and 2) randomisation of testing sequence of testing (section 5 and 8.3) |
| 13.10.21 | 2.3 | Correction in section 13.1 from ‘therefor 10 of the 40 patients’ to ‘therefore 8 of the 40 patients’ – in response to MHRA review. Approved by MHRA. |
| 01.11.21 | 3 | All changes requested by MHRA accepted. Approvals section updated (16.1). Additional personnel added. Membership of SSC updated (section 15). Reporting of reportable events to sponsor changed to 3 days rather than 24 hours in line with their SOP. |

Clinical Evaluation of a Novel LiveOCT Device to Improve the Management of Eye Disease

Version: 3

IRAS ID: 292051

Sponsor: Liverpool University Hospital NHS Foundation Trust, joint with The University of Liverpool

Study Protocol

Study Personnel

Clinical Queries should be directed to professor S.B. Kaye

| *Chief/ Principal Investigator* | Stephen Kaye, BSc, *MBBch, MD, FRCS, FRCOphth* | Consultant Ophthalmologist, Professor of Ophthalmology, Institute of Life Course and Medical Sciences, University of Liverpool and The Liverpool University Hospitals NHS Foundation Trust, 8Z Link, Prescot Street, Liverpool L7 8XP. |
| --- | --- | --- |
|  |  |  |
| *Co-Investigators* | Vito Romano  *MD, PGDip CRS, MHA*  Colin Willoughby  *BSc (Hons), FRCOphth, MD* | Consultant Ophthalmologist, Associate Professor of Ophthalmology, Institute of Life Course and Medical Sciences, University of Liverpool and The Liverpool University Hospitals NHS Foundation Trust, 8Z Link, Prescot Street, Liverpool L7 8XP.  Professor of Ophthalmology, School of Biomedical Sciences, Faculty of Health and Life Sciences, University of Ulster, Cromore Road, Coleraine, Co. Londonderry, BT52 1SA |
|  |  |  |
|  | Simon Harding *MB ChB, FRCS, FRCOphth, MD* | Chair Professor of Clinical Ophthalmology  Institute of Life Course and Medical Sciences, University of Liverpool William Henry Duncan Building, 6 West Derby Street, Liverpool, L7 8TX |
|  |  |  |
|  | Yaochun Shen  *PhD*  Yalin Zheng  *PhD*  Rhiannon Tudor *Edwards, BScEcon, MA.Econ, DPhil* | Professor of Electrical Engineering and Electronics, Department of Electrical Engineering and Electronics, University of Liverpool, L69 3GJ  Reader in Ophthalmic Imaging,  Institute of Life Course and Medical Sciences, University of Liverpool William Henry Duncan Building, 6 West Derby Street, Liverpool, L7 8TX  Professor in Health Economics, School of Health Sciences, Bangor University, Bangor, Gwynedd, LL57 2DG |
|  |  |  |
|  |  |  |
|  |  |  |
| *Study Staff* | Sharon Mason  *BSc, MSc, PhD* | Project Manager, Institute of Life Course and Medical Sciences, University of Liverpool William Henry Duncan Building, 6 West Derby Street, Liverpool, L7 8TX |
|  |  |  |
|  | Samuel Lawman  *BSc, PhD* | Post-Doctoral Research Associate, Department of Electrical Department of Electrical Engineering and Electronics, University of Liverpool Liverpool, L69 3GJ |
|  | Alex Undan (Study Nurse) | The Liverpool University Hospitals NHS Foundation Trust, 8Z Link, Prescot Street, Liverpool L7 8XP. |
|  | Andrea Madden (Study Nurse) | The Liverpool University Hospitals NHS Foundation Trust, 8Z Link, Prescot Street, Liverpool L7 8XP |
|  | Mohammed Ahmad  FRCOphth | The Liverpool University Hospitals NHS Foundation Trust, 8Z Link, Prescot Street, Liverpool L7 8XP |
|  | Kunal Gadhvi  FRCOphth  Luca Pagaona  MD  Adela Hulpus  FRCOphth  Mahmoud Ahmad  FRCOphth | The Liverpool University Hospitals NHS Foundation Trust, 8Z Link, Prescot Street, Liverpool L7 8XP  The Liverpool University Hospitals NHS Foundation Trust, 8Z Link, Prescot Street, Liverpool L7 8XP  The Liverpool University Hospitals NHS Foundation Trust, 8Z Link, Prescot Street, Liverpool L7 8XP  The Liverpool University Hospitals NHS Foundation Trust, 8Z Link, Prescot Street, Liverpool L7 8XP |

Table of Contents

[List of abbreviations 1](#_Toc86830217)

[1 Study summary 2](#_Toc86830218)

[2 Background 3](#_Toc86830219)

[3 Rationale 3](#_Toc86830220)

[4 Objectives and outcome endpoints/measures 3](#_Toc86830221)

[4.1 Objectives 3](#_Toc86830222)

[4.1.1 Primary objective 3](#_Toc86830223)

[4.1.2 Secondary objectives 4](#_Toc86830224)

[5 Study design 4](#_Toc86830225)

[6 Study setting 6](#_Toc86830226)

[7 Study participants 6](#_Toc86830227)

[7.1 Overall description of study participants 6](#_Toc86830228)

[7.2 Inclusion criteria 6](#_Toc86830229)

[7.3 Exclusion criteria 6](#_Toc86830230)

[8 Study procedures 6](#_Toc86830231)

[8.1 Screening and eligibility assessment 6](#_Toc86830232)

[8.2 Informed consent 6](#_Toc86830233)

[8.3 Baseline and subsequent assessments 7](#_Toc86830234)

[9 Study treatments 9](#_Toc86830235)

[9.1 Description of study intervention(s) 9](#_Toc86830236)

[9.2 Maintenance and storage of device 9](#_Toc86830237)

[10 Proposed outcome measures 9](#_Toc86830238)

[10.1 Primary outcomes 9](#_Toc86830239)

[10.2 Secondary outcomes 9](#_Toc86830240)

[11 Definition of end of study 10](#_Toc86830241)

[12 Discontinuation/ withdrawal of participants from study treatment 10](#_Toc86830242)

[13 Statistics and data analysis 10](#_Toc86830243)

[13.1 Sample size calculation 10](#_Toc86830244)

[13.2 Statistical analysis plan 11](#_Toc86830245)

[13.3 Economic evaluation 11](#_Toc86830246)

[14 Data management 13](#_Toc86830247)

[14.1 Data collection tools and source document identification 13](#_Toc86830248)

[14.2 Access to data 14](#_Toc86830249)

[14.3 Archiving 14](#_Toc86830250)

[15 Monitoring 14](#_Toc86830251)

[16 Ethical and regulatory considerations 15](#_Toc86830252)

[16.1 Approvals 15](#_Toc86830253)

[16.2 Adverse event and safety reporting 15](#_Toc86830254)

[17 Financing and insurance 18](#_Toc86830255)

[18 Peer review 18](#_Toc86830256)

[19 Audits 18](#_Toc86830257)

[20 Dissemination and intellectual property 18](#_Toc86830258)

[References 19](#_Toc86830259)

List of abbreviations

ADE Adverse Device Effect

AE Adverse Event

CERC Clinical Eye Research Centre

CRF Case Report Form

ECD Endothelial cell count

FMEA Failure Modes and Effects Analysis

GCP Good Clinical Practice

FECD Fuch’s Endothelial Corneal Dystrophy

LIVE-OCT Liverpool Optical Coherence Tomography

LUHFT Liverpool University Hospitals NHS Foundation Trust

OCT Optical Coherence Tomography

PIS Patient Information sheet

REC Research Ethics Committee

SADE Serious Adverse Device Effects

SAE Serious Adverse Event

SPEU St Paul’s Eye Unit

UoL University of Liverpool

USADE Unanticipated Serious Adverse Device Effect

1. Study summary

| Study Title | Clinical Evaluation of a Novel LiveOCT Device to Improve the Management of Eye Disease | |
| --- | --- | --- |
| Internal ref. no. (or short title) | LiveOCT Study | |
| Clinical Phase | Na | |
| Study Design | Prospective Observational Study | |
| Study Participants | Patients aged 12 years and above with a diagnosis of either keratoconus or Fuchs endothelial corneal dystrophy (FECD) and/ or corneal lamellar surgery and subjects with no corneal disease (healthy volunteers). | |
| Planned Sample Size | 40 patients with keratoconus, 30 with FECD and/or corneal lamellar surgery, and 20 with no known corneal abnormalities (healthy volunteers). | |
| Treatment duration | N/A | |
| Follow up duration | Baseline and follow up 3 months and 6 months | |
| Planned Study Period | *1^st^ May 2021 to 30^th^ Oct 2022* | |
|  | Objectives | Outcome Measures |
| Primary | To compare image quality | The quality of the LiveOCT images from 2 variants (D1 and D2) will be compared with the existing models according to predefined diagnostic image criteria. |
| Secondary | To assess repeatability of image quality  Sensitivity  Assess cost effectiveness  Patient experience |  |
| Medical device | Liverpool Optical Coherence Tomography device (LiveOCT). | |
| Formulation, Dose, Route of Administration | Na | |

1. Background

Optical coherence tomography (OCT) has been widely used in the management of eye disease. Commercially available OCT systems are still unable to meet the increasing demand posed by clinical decision-making. With an i4i grant, we have in Liverpool invented a new OCT device. This has enabled us to achieve in vitro in human corneas an improved resolution down to 2.1 microns. While the OCT device worked well in its current form in the laboratory, we were to yet translate this application into clinical practice and potentially provide benefit to patients. This translation required relevant in vivo device optimisation and improvement as well as clinical studies to demonstrate its potential to help improve the management of ophthalmic diseases.

1. Rationale

In our current i4i project (Ref: II-LA-1116-20008), we are planning to address this by realising a novel clinically proved OCT device that makes use of new technologies and approaches that can be used on human subjects. Having designed and built the device, we hope to evaluate and optimise the device and data analysis on both healthy volunteers and patients with various cornea diseases. The project will ultimately bring significant benefits for people with eye disease, improving healthcare, and subsequently reducing social and economic costs. We have developed a proof-of-concept observational study protocol to demonstrate clinical utility for diagnosis, disease classification and management. We will examine the performance of our LiveOCT device with comparison to current clinically used devices (corneal topography and tomography devices including OCT and ultrasound) with particular regard to intra, inter-observer and inter-device variability. This will be used to determine precision and accuracy, the latter based on currently accepted clinical standards. In comparison with other standard techniques, we hope to identify the uniqueness of our LiveOCT for supporting improved diagnosis, monitoring and treatment of eye diseases. We have developed 2 slightly different variants of our LiveOCT device, device 1 (D1) and device 2 (D2). The only difference between the variants is a very slight modification of the internal optics, which results in different image properties. There will be no difference in operation or patient experience between the variants.

1. Objectives and outcome endpoints/measures

## Objectives

### Primary objective

- To compare the quality and information provided of corneal images obtained with the new LiveOCT device variants (D1 and D2) with those obtained using standard clinical instruments including existing OCT devices.

### Secondary objectives

- To evaluate repeatability, sensitivity and specificity of the LiveOCT device.
- To evaluate costs and consequences- from an NHS perspective we will explore the costs and consequences of the LiveOCT device as compared to usual care.
- To study patient experience on LiveOCT device.

1. Study design

This study will follow a prospective observational study design. The single study site is St. Paul’s Eye Unit, The Liverpool University Hospitals NHS Foundation Trust (LUHFT).

Following recruitment, eligibility assessments and consent, participants will undergo a baseline assessment. Follow up assessments will take place after 3 and 6 months.

During each visit, the participant will undergo standard eye examinations (visual acuity and slit-lamp bio microscopy), and non-invasive tests including endothelial cell counting (ECD), tomography, and OCT imaging by standard OCT devices (TOMEY CASIA SS-1000 and Heidelberg Spectralis) and our new LiveOCT device variants (D1 and D2). Two qualified individuals, both operating one of two LiveOCT devices equipped with the study software, will perform the study device measurements. Each patient will be measured three times with each device at both the baseline and at follow-up visits (after 3 and 6 months). See figure 1 for the study design showing the study design/ patient pathway.

We expect the duration of D1 and D2 assessments (capturing 3 images on each device per visit) to take 15 minutes for each device, so 30 mins in total will be dedicated to our novel OCT devices each visit. The order of the OCT devices will be randomised using a random permutation approach to avoid bias through patient or investigator fatigue but the all measurements on each device will take place at the same time as to avoid delays caused by positioning the patient.

   
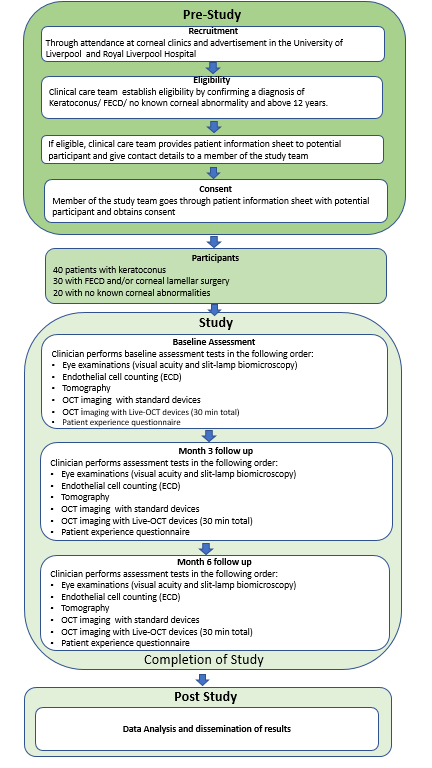


Figure 1. Schematic of the Study design/ patient pathway

1. Study setting

The Study will run in a single site at the Clinical Eye Research Centre (CERC) in St. Paul’s Eye Unit within The Liverpool University Hospitals NHS Foundation Trust (LUHFT).

1. Study participants

## Overall description of study participants

90 subjects 12 years old and above who meet the inclusion and exclusion criteria will be recruited and subsequently tested at the cornea clinics of St Paul’s Eye Unit (SPEU), LUHFT. There will be 3 groups of participants: 40 patients with keratoconus, 30 with FECD and/or corneal lamellar surgery, and 20 with no known corneal abnormality.

## Inclusion criteria

Patients, male or female, aged 12 years and above with a diagnosis of either keratoconus or Fuchs endothelial corneal dystrophy (FECD)/ corneal lamellar surgery and subjects with no corneal disease (healthy volunteers).

## Exclusion criteria

In this study, patients who have poor fixation or have a learning disability will not be excluded as this will be an opportunity to hopefully demonstrate the advantage of our new LiveOCT device. Exclusion criteria: Nystagmus, under 12 years old, inability to provide informed consent,

1. Study procedures

## Screening and eligibility assessment

Eligible patients and their guardians (if appropriate) attending the corneal clinics, will initially be approached by members of the direct clinical care team and given an A4 advert for our study. If interested, a simple eligibility assessment will be carried out. If eligible, they will be asked if they would be willing to be contacted by a member of the study team to discuss further. If agreeable, they will be given the participant information sheet (PIS) and told they will be contacted subsequently by an investigator.

## Informed consent

When contacted, potential participants will be taken through the information sheet and given opportunity to ask questions. Written and verbal versions of the participant information and Informed consent will be presented to the participants detailing no less than: the exact nature of the study; the implications and constraints of the protocol; the known side effects and any risks involved in taking part. It will be clearly stated that the participant is free to withdraw from the study at any time for any reason without prejudice to future care, and with no obligation to give the reason for withdrawal.

For people with learning disabilities their legal guardian will be asked to witness and or give consent on their behalf after they have shown they have understood the information provided in the PIS and consent form.

The decision to include children (12 years old and above) in this study was made because this is the age when Keratoconus may start and we are hoping to observe the initial changes caused by this condition. The legal guardian/ parent of the child will be asked to give consent on their behalf after they have shown they have understood the details provided in the PIS and consent form. The child will be involved in the decision-making process as much as possible and will be asked to give assent.

The participant will be allowed up to 2 weeks to consider the information and the opportunity to question the Investigator, their GP or other independent parties to decide if they would like to participate in the study. Written Informed Consent will then be obtained by means of participant dated signature and the dated signature of the person who presented and obtained the informed consent, using the latest approved version of the informed consent form. The person who obtained the consent must be suitably qualified and experienced and have been authorised to do so by the Chief Investigator according to the ethically approved protocol, principles of Good Clinical Practice (GCP) and Declaration of Helsinki. A copy of the signed Informed Consent will be given to the participants. The original signed form will be retained at the NHS research site.

The study will be carried out by following UoL and LUHFT guidance and policy associated with COVID-19, as well as the Government’s COVID-19 guidance and law. Social distancing and face covering guidelines will be followed as appropriate to keep both researchers and participants safe. All participants will be recruited from scheduled appointments at the clinics at St Paul’s Eye Unit.

## Baseline and subsequent assessments

For each participant, there will be 3 visits: baseline, month 3 and month 6. During each visit, the participant will undergo standard eye examinations (visual acuity and slit-lamp bio microscopy), and non-invasive tests including endothelial cell counting (ECD), tomography, and OCT imaging by standard OCT devices (TOMEY CASIA SS-1000 and Heidelberg Spectralis) and our new LiveOCT device variants. Two qualified individuals, both operating one of two LiveOCT devices equipped with the study software, will perform the study device measurements. Each patient will be measured three times with each device at both the baseline and at follow-up visits. A questionnaire will be completed on their experience and view of the device at the end of each visit.

The following procedures will be carried out on each patient at baseline, month 3 and month 6, as shown in Table 1:

1. Eye examinations (visual acuity and slit-lamp bio microscopy)
2. Endothelial cell counting (ECD) with confocal microscopy
3. Tomography with Pentacam camera
4. OCT imaging with devices currently in clinic (The TOMEY Casia and Heidelberg Spectralis)
5. Imaging by our new LiveOCT device – variant 1 (D1) and 2 (D2)
6. Patient experience questionnaire

The order of the OCT devices will be randomised using a random permutation approach to avoid bias through patient or investigator fatigue but the all measurements on each device will take place at the same time as to avoid delays caused by positioning the patient.

All the procedures except for imaging with our new LiveOCT device are part of standard care. For patients with keratoconus, all the three visits are standard patient care pathway. For patients with FECD, month 3 visit is an additional visit to the standard care. For healthy volunteers, all the 3 visits will be research activities.

**Table 1: Tests undertaken during each of the three visits.**

|  | **Baseline (month 0)** | **Month 3 Visit** | **Month 6 visit** | **Comments** |
| --- | --- | --- | --- | --- |
| **Eye examination (visual acuity and slit lamp bio microscopy)** | V | V | V | Standard Clinical procedure |
| **Endothelial cell counting (ECD)** | V | V | V | Standard Clinical procedure |
| **Tomography** | V | V | V | Standard Clinical procedure |
| **Tomography** | V | V | V | Standard Clinical procedure |
| **Imaging using our new LiveOCT devices, D1** | V | V | V | Extra test |
| **Imaging using our new LiveOCT device, D2** | V | V | V | Extra test |
| * All the procedures except for imaging by using our new OCT device are part of standard care pathway  * For patients with Keratoconus, all the three visits are standard patient care pathway  * For patients with FECD, month 3 visit is an additional visit to the standard care  * For healthy volunteers, all 3 visits will be research activities | | | | |

1. Study treatments

## Description of study intervention(s)

*In vivo* non-contact imaging of the layers of the cornea using a new anterior segment LiveOCT imaging device. 2 slightly different variants will be used. The only difference between variant 1 (D1) and variant 2 (D2) will be a small modification to the internal optics with no difference to user or patient experience.

## Maintenance and storage of device

The device will be stored in the study room, which will be locked when not in use. It does not need to be kept in special conditions. The device will be monitored and maintained by the team throughout the clinical study.

1. Proposed outcome measures

## Primary outcomes

**Image quality**: The quality of the LiveOCT images will be compared with the existing models according to predefined diagnostic image criteria.

**Added information:** The anticipated additional information provided by our LiveOCT will then be used to determine if and what changes have occurred in the patients with FECD and keratoconus that were not apparent with the standard tests and whether these changes reflect or indicate change (deterioration or improvement) in the status of the disease. We will also determine which additional components of the cornea have been affected and whether and to what extent are the features reproducible. An evaluation will also be undertaken to determine whether the information provided by our LiveOCT is associated with specific features evident in the images from the standard instruments and whether the information is new or redundant.

## Secondary outcomes

**Repeatability/reproducibility:** Outcome metrics will be the repeatability and reproducibility of measurements (identification and thickness) of structural parameters of the cornea, corneal surface contour and refractive power.

**Sensitivity and specificity:** We will use published grading systems specific to the two main conditions of FECD and keratoconus. For keratoconus, the sensitivity of the LiveOCT will be assessed by its ability to detect the same diagnostic parameters of non-uniform and focal corneal thinning and irregular and non-asymmetric anterior and posterior corneal profiles currently measured using the Pentacam. For FECD, the sensitivity of the LiveOCT will be assessed by its ability to measure increases in corneal thickness and areas of corneal swelling, and excrescences (guttata) and thickening of Descemet's membrane in patients who have guttata evident with slit lamp bio microscopy and increased corneal thickness measured with the Pentacam. Specificity will be defined as the percentage in which the described diagnostic changes in the cornea for keratoconus and FECD are not apparent with the LiveOCT device.

**Patient experience:** Study participants will be asked to fill in a simple set of questions concerning their experience with the LiveOCT devices. The patients will be asked to fill out paper copies of the questionnaire after the end of each study appointment, but the study nurse will be available to help if required.

If the participant indicates that they are feeling particularly anxious or depressed on the questionnaire, a member of their care team will pass this information onto their GP.

**Cost Effectiveness:** See 13.3 - economic evaluation

1. Definition of end of study

The study will be completed and terminated on 31st October 2022, which is defined to be the date when analysis of the data collected during the trial is being completed.

Suspension or early termination of the study when there are serious concerns about subjects’ safety and inadequate performance, that cannot be mitigated by modifications of the study protocol based upon the review of the safety data.

1. Discontinuation/ withdrawal of participants from study treatment

If a decision is made by the participant and or/clinician not to continue with the study, the participant will be removed from it and the event recorded. Data will still be collected unless the participant explicitly withdraws consent for it.

1. Statistics and data analysis

## Sample size calculation

The current gold standard for measuring the cornea is based on the tomographic images of the Pentacam for the thinnest corneal thickness (TCT). There is insufficient evidence in the literature to determine what change might occur in Bowman’s layer longitudinally. Bowman’s layer has not been measured before using a Pentacam so that we do not have a comparison. The primary endpoint, therefore, is a change in TCT. We do not know whether the change in TCT will be reflected in a change in Bowman’s layer and or Descemet’s membrane thickness and therefore we are not able to use the changes in these layers to determine the sample sizes. The changes in Bowman’s layer and Descemet’s membrane will be exploratory but we expect that changes will occur as TCT changes. For TCT, using a Pentacam, the cut-off for a change in TCT is 12.1um between the mean of three measurements on 2 occasions (Brunner et al, 2018). We expect the LiveOCT to detect all patients who have this change, that is, >12.1um in TCT. The incidence of patients with keratoconus (KC) changing over a 6 month period is expected to be 20% using the Pentacam. Therefore 8 of the 40 patients included will be expected to show this change using the Pentacam. Based on the increased resolution of the Liverpool LiveOCT we expect a much greater sensitivity for detecting change in TCT. We estimate that 50% of patients will show a change that we can detect based on the increased resolution of 2μm of the LiveOCT compared to 20μm of the Pentacam. Evidence for the current OCT performed worse than the Pentacam (Brunner et al) hence the choice of the Pentacam as a comparison for TCT. A difference, therefore, of between 20% and 50% would require a sample size of 36 with an alpha of 0.05 and beta 0.2. Given that over the course of the study 10% will drop out we calculate a sample size of 40 patients with KC. For patients with FECD, we do not have data on the change in TCT, however, extrapolating from patients with KC the change in TCT is usually greater in FECD hence a similar or slightly smaller sample size is needed which we estimate to be 30 patients. For healthy subjects we do not expect the change in TCT to be greater than the 95% TCT cut-off 8.81um (Pentacam) over the course of the study, based on Brunner et al 2018. We, therefore, expect 20 subjects in this group to provide reproducibility data using the LiveOCT.

## Statistical analysis plan

Statistical analysis will be performed as appropriate using SPSS version 20.0 (SPSS IBM, Chicago, IL, USA). For example, the Bland-Altman analysis will be used to visualise agreement, the coefficients of variation (CV) analysis will be performed to assess repeatability and reproducibility, and two-way random effect ANOVA model (REML) will be used to assess the random effects operator (confounded with device) and eye for the measured endpoints were within the specified range for this device. Sensitivity and specificity tests will be performed.

## Economic evaluation

Data on health economics will be analysed by the team at Bangor University led by Professor Rhiannon Tudor Edwards. From an NHS perspective, we will undertake a cost-consequences analysis presenting the outcomes and cost in disaggregated form. We will collect outcome data (e.g. scores of quality corneal images and length of acquisition time of the new OCT device and the existing OCT device) for the 3 groups of participants at baseline, month 3 and month 6. Liaising with The LUHFT staff, we will calculate the cost of performing eye test per visit per participant using our LiveOCT device and the existing OCT device (if necessary) respectively. Lessons learnt from this observational study will provide guidance to inform further research including a plan for an economic evaluation alongside a future randomised controlled trial.

**Micro-costing analysis**

We will undertake a micro-costing of the introduction of the LiveOCT device into clinic based on our previous use of micro-costing. We will explore what there is to be learnt from recent developments in micro-costing in health economics and in time-driven activity-based costing as part of the value-based health care movement.

In order to undertake our cost-consequence analysis we will collect data at baseline, 3 and 6 months. See table 2.

**Table 2:** Draft plan for preliminary health economics data collection

| **No.** | **Data** | **Details** |
| --- | --- | --- |
| **Intervention delivery data** | | |
| 1. | Staff | Type of staff (i.e. type of healthcare professional), number & grade of staff who prepare, conduct the imaging, analyse scan, provide feedback to patients regarding scan and present in the imaging room for each patient’s OCT imaging appointment for both standard and new OCT imaging at each study time-point. |
| 2. | Duration of time taken for… | Preparatory, administration, conducting the imaging, analysis of the scan and feeding back to patients regarding scan for each patient’s OCT imaging appointment for both standard and LiveOCT imaging at each study time-point. |
| 3. | Disposable items | What are the disposable items and total quantity of each item for both standard and new OCT imaging? |
| 4. | Overheads | What are the relevant overheads to be charged for the department/hospital for both standard and new OCT imaging? |
| **Outcome measures -** | | |
| 5. | Primary - Quality corneal images score | What is the score of quality corneal images for each patient’s corneal image produced by standard OCT device and LiveOCT device at each study time-point? |
| 6. | Secondary – Patient’s experience, staff experience | We are aware that the main clinical study is collecting data on these. |

We will undertake deterministic sensitivity analysis to explore implications of for example various costs of the LiveOCT device and any time and motion implications of any additional time in clinic taken relating to the introduction of the LiveOCT device.

1. Data management

## Data collection tools and source document identification

All activities will be in line with local practices, ICH GCP, the Caldicott Guardian and the Data Protection Act 2018, UK’s implementation of the General Data Protection Regulation (GDPR).

All research subjects will be pseudo-anonymised by assigning a unique study ID to index all the data. Data that are collected for each research subject constitute correspondingly his/her CRF (Case Reports Form), which will cover the following areas/topics:

• Inclusion criteria and consent

• Demographics

• Medical history

• Medication

• Surveys and questionnaires

• Adverse events

• Binary files (images)

At the end of each appointment the study nurse will check the CRFs for completeness before the participant leaves so that tests can be completed if required. If an omission is noticed after the participant has left they will be asked to return if reasonably possible within a week of the initial appointment. Any major corrections or major missing data such as age, gender, type of disease, images not taken, incorrect recording of visual acuity will be noted and corrected by the research nurse.

Only NHS research staff working on this project will have access to the patient identifiable data. No patient identifiable data will be transferred out of the Trust. Only pseudonymised data will be transferred out of the Trust (transferred using encrypted portable hard drive to the University research team for analysis).

For the new LiveOCT device, data will be collected by the device software on its PCs device. The encrypted data will be synchronised between LiveOCT devices and an encrypted external hard drive and backed up using a dedicated external hard drive every evening. The external hard drives will be kept in a secure location. The PCs will also have a windows user account access control. For all stored data, raw image data will not be encrypted but not stored in a manner identifiable to a patient. Encrypted note files will be created for each “visit” but not stored in a manner identifiable to a patient. An encrypted database will link image data and encrypted notes to patients, and store patient identifiable details (only names for validating patient ID in use). Each participant will be assigned an anonymous study number. Project software will be required to read encrypted database data and files and software access will be controlled by valid username and password access. User names will only be issued to authorised project members and staff employed on the clinical study. Software can produce unencrypted pdf reports of individual patients for clinical purposes if needed. For study purposes, export of data for analysis has patient identifiable data (excluding study ID) and notes removed. The spreadsheets containing the data will be locked every 3 months. All exported data will further be secured on encrypted drives only.

For further information please see our comprehensive data management plan and other associated documents (referred to in our data management plan).

## Access to data

Identifiable information will only be accessed by the authorised research team at the NHS research site. Any participant information (e.g. images and questionnaires) will be pseudo-anonymised within the LUHFT before being transferred to the universities for analysis, and stored securely at the university’s secured network drive. Pseudo anonymised data related to health economics analysis will be shared with Prof Rhiannon Tudor Edwards at Bangor University.

## Archiving

Data collected, required software to read and associated analyses will be deposited in a data archive at the end of the project and stored for 15 years.

Frequent backups are performed using the best enterprise backup solutions at LUHFT as well as UoL, and are physically stored in a fire-proof safe. The backup strategy will be implemented to support hourly, daily, monthly and yearly retention.

1. Monitoring

Study monitoring will be carried out to ensure that the rights and well-being of the participants are protected during the study. A risk assessment will be performed prior to initiation. This will be completed in partnership with representatives of the study sponsor, the Chief Investigator, the Clinical Lead and the Study Manager.

The study will be managed by an Executive group, which will meet face-to-face 3 to 4 times per year and by teleconference approximately fortnightly. The Executive group for the study will be chaired by the Chief Investigator and will consist of the applicants and the trial manager.

The study will be overseen by a study steering committee that will be convened before the start of recruitment.

Membership of the study steering committee will include:

- Independent chair ( Ms. Nardine Menassa, Consultant Ophthalmologist at The Royal Liverpool University Hospital)
- Independent Patient representative (Diana Perry, ED Society)
- Independent Clinical Representative (Ms Teresa Sandinha, Consultant Ophthalmologist at The Royal Liverpool University Hospital)Study representative (Prof Stephen Kaye, Chief Investigator) – non independent member

The steering committee will check that we are adhering to protocols and recording data and adverse events appropriately and accurately at month 3 and month 6 of the study.

1. Ethical and regulatory considerations

## Approvals

## All approvals (NRES, MHRA Notice of No Objection and HRA) will be in place before we commence recruitment. Adverse event and safety reporting

*The research team do not expect there will be any AE or SAE, however, in the event they do occur, the procedures below will be followed.*

Definitions from the ‘Guidelines on Medical Devices’ from the European Commission (‘Clinical investigations: serious adverse event reporting under directives 90/385/EEC and 93/42/EEC’) and the examples that would apply to the LiveOCT study are presented in the table below

| **Term** | **Definition** | **Example(s) applying to LiveOCT.** |
| --- | --- | --- |
| Adverse Event (AE) | Any untoward medical occurrence, unintended disease or injury or any untoward clinical signs (including an abnormal laboratory finding) in subjects, users or other persons whether or not related to the investigational medical device.  NOTE 1: This definition includes events related to the investigational device or the comparator.  NOTE 2: This definition includes events related to the procedures involved.  NOTE 3: For users or other persons, this definition is restricted to events related to investigational medical devices. | Irritation of forehead chin/ hands in contact with device.  Irritation of eye from Laser  Trapping of fingers in device |
| Serious Adverse Event (SAE) | Adverse event that:  a) led to a death, injury or permanent impairment to a body structure or a body function.  b) led to a serious deterioration in health of the subject, that either resulted in:  - a life-threatening illness or injury, or  - a permanent impairment of a body structure or a body function, or  - in-patient hospitalization or prolongation of existing hospitalization, or  - in medical or surgical intervention to prevent life threatening illness  c) led to foetal distress, foetal death or a congenital abnormality or birth defect.  NOTE 1: Planned hospitalization for pre-existing condition, or a procedure required by the Clinical Investigation Plan, without a serious deterioration in health, is not considered a serious adverse event. | Serious electrocution  Serious injury of eye from laser |
| Device Deficiency | Inadequacy of an investigational medical device related to its identity, quality, durability, reliability, safety or performance. This may include malfunctions, use error, or inadequacy in the information supplied by the manufacturer. | Device presents misleading information |
| Adverse Device Effect (ADE) | Adverse event related to the use of an investigational medical device.  NOTE 1: This includes any adverse event resulting from insufficiencies or inadequacies in the instructions for use, the deployment, the implantation, the installation, the operation, or any malfunction of the investigational medical device.  NOTE 2: This includes any event that is a result of a use error or intentional abnormal use of the investigational medical device. | As device deficiency  Patient face knocked with device whilst aligning. |
| Serious Adverse Device Effects (SADE) | Adverse device effect that has resulted in any of the consequences characteristic of a serious adverse event. | As SAE.  Device falls onto patient requiring hospitilisation |
| Unanticipated Serious Adverse Device Effect (USADE) | Serious adverse device effect which by its nature, incidence, severity or outcome has not been identified in the current version of the risk analysis report.  NOTE: Anticipated SADE (ASADE): an effect which by its nature, incidence, severity or outcome has been previously identified in the risk analysis report. | Photo induced epileptic seizure. |

The CI will investigate all adverse events and reactions and grade and record them in the patients CRF. Any patient who has an adverse reaction will be referred to either the Accident and Emergency Department or the St. Paul’s Primary Eye Care Department of the RLUH within LUHFT or to their General Practitioner depending on the situation. The monitoring of the reporting of adverse events and reactions will be undertaken by a study steering committee chaired by Consultant Ophthalmologist, Mr Nardine Menassa

**Reported by investigator to sponsor**

The CI of the study will report the reportable events to the sponsor not later than 3 calendar days after investigational site study personnel’s awareness of the event.

**Reported by sponsor to MHRA**

Reportable events will be reported at the same time to the MHRA by the sponsor through the Yellow Card Scheme.

**Investigation**

The MHRA, the sponsor and the investigators of the study may investigate the problem depending on how serious it is.

**Causality assessment** The relationship between the use of the LiveOCT device and the occurrence of each adverse event shall be assessed and categorized. For the purpose of harmonising reports, each SAE will be classified according to five different levels of causality (MEDDEV 2.7/3 revision 3).
 • Not related
 • Unlikely
 • Possible
 • Probable
 • Causal relationship

The study will be halted immediately once any AE or SAE occurs.

Testing will be halted immediately if the participant becomes distressed or indicates that they wish testing to end, the authorised users will report the AE to the Chief Investigator (Prof Stephen Kaye)

1. Financing and insurance

The study is fully funded by a NIHR i4i Product Development Award. The NIHR Clinical Research Network North West Coast (CRN-NW) and The LUHFT have jointly attributed all activities associated with the clinical study using the AcoRD model and provided the NHS Treatment Costs, Service Costs and Research Costs.

1. Peer review

Thorough peer review was carried out during application to the funder for this research and this protocol was peer reviewed by Esmaeil Arbabi, consultant ophthalmologist at the Liverpool University Hospitals NHS Foundation Trust, and Professor Xujiong Ye at the University of Lincoln.

1. Audits

The study will be subject to inspection and audit by the sponsor (TBD) under their remit as the sponsor and other regulatory bodies to ensure adherence to GCP and the UK Policy Framework for Health and Social Care Research (v3.2 10th October 2017).

1. Dissemination and intellectual property

The dissemination activities of the study outcomes will be carefully planned to be compatible with the protection of IP. For the purposes of IP protection, during the project all publications, presentations, and other dissemination-related activities shall first be reviewed by Shona Jones and the IP team within the University, and the funder i4i Programme Secretariats. The new IP generated will be protected under the agreement between The University of Liverpool and Liverpool University Hospitals NHS Foundation Trust.

# References

1.Huang D, Swanson EA, Lin CP, et al. Optical coherence tomography. Science, 1991;254:1178-1181.

2.Drexler W, Fujimoto JG. State-of-the-art retinal optical coherence tomography. Prog Retin Eye Res, 2008;27:45-88.

3.Lawman S et al. High resolution corneal and single pulse imaging with line field spectral domain optical coherence tomography. Opt Express 2016;24(11):12395-405

4.Drummond M, Sculpher M, Torrance G, O’Brien B and Stoddart G. Methods for the Economic Evaluation of Health Care Programmes. Oxford: Oxford University Press, 2005.

5.Phillips CJ. Health Economics: An introduction for health professionals. Oxford: Blackwell Publishing Ltd, 2005.

6.Brazier J, Ratcliffe J, Salomon J and Tsuchiya A. Measuring and Valuing Health Benefits for Economic Evaluation. Oxford: Oxford University Press, 2007.

7.Yeo ST, Edwards RT, Luzio SD, Charles JM, Thomas RL, Peters JM, Owens DR. Diabetic retinopathy screening: perspectives of people with diabetes, screening intervals and costs of attending screening. Diabetic Med, 2012a, 29(7), 878-885.

8.Yeo ST, Edwards RT, Fargher EA, Luzio SD, Thomas RL, Owens DR. Preferences of people with diabetes for diabetic retinopathy screening: A discrete choice experiment. Diabetic Med, 2012b, 29(7), 869-877.

9. Brunner M, Czanner G, Vinciguerra R, et al. Improving precision for detecting change in the shape of the cornea in patients with keratoconus. Sci Rep, 2018;8(1):12345.

10. Charles, J. M., Edwards, R. T., Bywater, T., & Hutchings, J. Micro-costing in public health economics: steps towards a standardized framework, using the incredible years toddler parenting program as a worked example. Prevention Science, 2013, 14(4), 377-389.

11. Keel, G., Savage, C., Rafiq, M., & Mazzocato, P. Time-driven activity-based costing in health care: A systematic review of the literature. Health Policy, 2017, 121(7), 755-763.
